# Assessment of Mechanical Stress Induced by Radiofrequency Currents on Skin Interfaces

**DOI:** 10.1155/2021/6623757

**Published:** 2021-10-11

**Authors:** Ilja L. Kruglikov

**Affiliations:** Scientific Department, Wellcomet GmbH, 76229 Karlsruhe, Germany

## Abstract

The epidermal-dermal (ED) and dermal-subcutaneous (DS) junctions are the most prominent skin interfaces, which are known to be of primary importance in different dermatological and aesthetic conditions. These interfaces are strongly modified in aging skin, and their effective targeting can lead to improvement of skin appearance in aging and by cellulite. Application of radiofrequency (RF) currents to the skin can selectively produce mechanical stress on these interfaces. Here, we assess the stresses induced by RF currents of different frequencies on EDJ and DSJ and discuss possible applications of the interfacial therapy in aesthetic medicine.

## 1. Introduction

Skin wrinkling is one of the most prominent signs of aging skin and one of the most important targets in antiaging therapy. Mechanically, human skin appears as a multilayer composite containing the stiff thin cover layer of stratum corneum (SC) followed below by more compliant layers of viable epidermis and dermis and further below by much more compliant layer of subcutaneous white adipose tissue (sWAT). Recently, we have argued that skin wrinkling must be substantially dependent on the quality of the interfaces between the single skin layers, especially of the epidermal-dermal (ED) and dermal-subcutaneous (DS) junctions [1]. Both interfaces are not flat and demonstrate typical undulations, which are continuously modified in chronological aging. Reduction of EDJ undulations (known as *rete ridges* or *papillae*) is strongly pronounced in the aged skin, and its restoration correlates with visual skin improvement. On the other hand, strong DSJ undulations into the dermis are known as a hallmark of cellulite [1], and these morphological structures react to mechanical loading with decrease of their height and contact area [2].

Both EDJ and DSJ are not the imaginary boundaries: each of them consists of several cell layers and has cellular content and intercellular structures that differ from the adjacent skin layers. For EDJ, this is the papillary dermal layer, which in the facial skin has a thickness of 20-30 *μ*m and mechanically appears as much less stiff as the underlying reticular dermis [1]. The main cells in this layer are the papillary fibroblasts, which phenotypically and physiologically differ from reticular fibroblasts [3]. Papillary and reticular fibroblasts have different morphologies and proliferation rates and are distinctly involved in skin development, regeneration, and fibrosis and demonstrate different biomarkers of aging [4].

For DSJ, this is the dermal white adipose tissue (dWAT), which consists of several layers of dermal adipocytes and is located between reticular dermis and sWAT as well as around hair follicles [5]. dWAT layer can quickly react to different stimuli and continuously expands with chronological aging [6]. Such modifications directly influence the mechanical properties of the skin: skin stiffness in mice was shown to be inversely dependent on the dWAT thickness [7]. Recently, we have demonstrated that dermal adipocytes belonging to DSJ have high plasticity and undergo reversible processes of de- and redifferentiation as well as transdifferentiation in myofibroblasts [8]. These processes sufficiently modulate the local mechanical properties of the skin making them cyclic and hair follicle-dependent. Additionally, dermal adipocytes strongly contribute to the metabolic regulation of dermal fibroblasts, among others influencing their ability to produce collagen [6]; hence, modulation of DSJ can also influence the physiology and morphology of adjacent reticular dermal layer.

All these new results raise the question concerning the targeted modification of the skin interfaces to improve the general mechanical properties of the skin. Previously, we have discussed application of very high frequency ultrasound (VHF-US) and special radiofrequency (RF) currents for targeting skin interfaces. It was shown that VHF-US is able to induce selective thermomechanical stress on DSJ causing appearance of sufficient thermic flow through this interface, and that this effect is strongly frequency dependent [9]. Additionally, it was demonstrated that an optimized distribution of RF electrodes on the skin surface can several times increase heating of the skin interfaces [10].

Whereas RF currents mainly affect the skin through production of Joule heating, they can additionally induce mechanical stress on the skin interfaces if the electrical properties of two adjacent skin layers significantly differ from each other. However, this effect was so far utterly neglected in RF applications. Here, we assess the thermomechanical stresses induced by RF currents of different frequencies on the EDJ and DSJ interfaces and discuss whether these forces are high enough to modify the mechanical properties of the skin.

## 2. Theory of Mechanical Stress Induced by RF Current on Skin Interfaces

Radiofrequency (RF) currents belong to the state-of-the-art treatments in noninvasive and minimal invasive aesthetic medicine. Physically, RF current may be of any frequency from the range 20 kHz-300 GHz; however, devices applied in dermatology and aesthetic medicine mainly operate with frequencies between 1 MHz and 10 MHz. It is widely accepted that the main effect of RF current on the skin is connected to its Joule heating. Moreover, it was proposed that such RF-induced hyperthermia can cause collagen shrinkage, and that this phenomenon is mainly responsible for the skin tightening effect claimed to appear after RF treatments. Collagen shrinkage is indeed well known from such invasive RF procedure as capsulorrhaphy (tightening of a hyperlexic joint capsule by its thermal shrinking [11]); however, this effect cannot be realized in the majority of dermatological treatment procedures based on application of noninvasive RF currents since they induce just a mild or moderate hyperthermia in the skin.

Electrically, both skin and underlying adipose tissue are lossy dielectrics, which not just dissipate but also store the applied RF energy. Whereas energy dissipation generally leads to tissue heating, storage of RF energy provides tissue polarization caused by a redistribution of electrical charges in a treated area. Ratio between these components of RF current in a given tissue varies with frequency demonstrating a pronounced dispersion. The predominant role of Joule heating produced by RF current can be unfolded only when the conductive part of RF current in the tissue significantly exceeds its displacement part, which in turn is connected to dielectric properties of the tissue. On the other hand, appearance of a sufficient displacement current can result in induction of mechanical stress on the interfaces between adjacent layers and will be especially pronounced for such RF at which the adjacent skin layers demonstrate sufficiently distinct dielectric properties.

Following continuity equation is valid in tissue volume:

(1) $$\begin{array}{c} \nabla \overset{⟶}{J}+\frac{\partial \rho }{\partial t}=0. \end{array}$$

Here, $\overset{⟶}{J}$ and *ρ* are the current and charge density, respectively. For an alternative current, $\overset{⟶}{J}$ can be written in a general form

(2) $$\begin{array}{c} \overset{⟶}{J}=\left[\sigma \left(f\right)+\frac{\partial }{\partial t}\varepsilon_{0}\varepsilon \left(f\right)\right]\overset{⟶}{E}=\left[\sigma \left(f\right)+i2\pi f\varepsilon_{0}\varepsilon \left(f\right)\right]\overset{⟶}{E}. \end{array}$$

Here, $\overset{⟶}{E}$ is the local strength of electric field in the skin, V/m; *f* is the current frequency, Hz; *σ*(*f*) is the electrical conductivity of the tissue at frequency *f*, S/m; *ε*_0_ = 8.854 × 10^−12^ F/m is the free space permittivity; *ε*(*f*) is the relative permittivity of the tissue at frequency *f*; $i=\sqrt{-1}.$ The first (conductive) term of the current density in (2) is responsible for energy dissipation and Joule effect in the tissue; the second (displacement) term is responsible for energy storage and tissue polarization.

On the interface between two lossy dielectric layers 1 and 2, Equation (1) can be transformed into

(3) $$\begin{array}{c} \overset{⟶}{n}\left(\overset{⟶}{J_{1}}-\overset{⟶}{J_{2}}\right)+\frac{\partial \rho_{s}}{\partial t}=0. \end{array}$$

Here, $\overset{⟶}{n}$ is the unit vector normal to the interface and directed from layer 2 into layer 1, and *ρ*_*s*_ is the surface charge density on this interface. Since $\rho_{s}=\overset{⟶}{n}\varepsilon_{0}\;\left(\varepsilon_{1}\overset{⟶}{E_{1}}-\varepsilon_{2}\overset{⟶}{E_{2}}\right)\;$ and $\overset{⟶}{J}=\sigma \overset{⟶}{E}$, the following boundary conditions for the tangential, *E*_*t*_, and normal, *E*_*n*_, components of electric field strength, $\overset{⟶}{E}$, must be valid on the interface between two lossy dielectrics:

(4) $$\begin{array}{c} E_{t,1}=E_{t,2}, \end{array}$$

(5) $$\begin{array}{c} \left(\sigma_{1}E_{n,1}-\sigma_{2}E_{n,2}\right)+\varepsilon_{0}\frac{\partial }{\partial t}\left(\varepsilon_{1}E_{n,1}-\varepsilon_{2}E_{n,2}\right)=0, \end{array}$$

with *E*_*i*_^2^ = *E*_*t*,*i*_^2^ + *E*_*n*,*i*_^2^; here, *E*_*i*_, *σ*_*i*_, and *ε*_*i*_ are the electric field strength, conductivity, and relative permittivity in layer *i*, respectively. For RF current with a frequency *f*, Equation (5) can be rewritten in the form *ε*_*T*1_*E*_*n*,1_ = *ε*_*T*2_*E*_*n*,2_, where *ε*_*Ti*_ = *ε*_0_*ε*_*i*_ − *jσ*_*i*_/*ω* is the complex permittivity and *ω* = 2*πf*.

The density of stored electric energy connected to displacement current can be presented in the form Ust=1/2ReεTE⟶2 with the time average value of Ust¯=1/4ReεTE⟶2; the density of dissipated energy and can be written as Udis=1/2ImεTE⟶2 with the time average value of Udis¯=1/4ImεTE⟶2. The total energy density in the layer *i* is

(6) $$\begin{array}{c} U_{i}=\frac{1}{2}\left(\varepsilon_{0}\varepsilon_{i}-j\frac{\sigma_{i}}{\omega }\right){\left|\overset{⟶}{E_{i}}\right|}^{2}. \end{array}$$

The absolute value of the total energy density |*U*_*i*_| describes the mechanical stress (pressure) produced by the normal component of applied electric field [12, 13].

(7) $$\begin{array}{c} p_{n,i}=\left|U_{i}\right|=\sqrt{U_{i}U_{i}^{*}}=\frac{1}{2}\sqrt{\varepsilon_{0}^{2}\varepsilon_{i}^{2}+\frac{\sigma_{i}^{2}}{\omega^{2}}}E_{n,i}^{2}. \end{array}$$

Here, *U*_*i*_^∗^ is the complex conjugate of *U*_*i*_.

The resultant additional pressure on the interface between two lossy dielectric layers produced by the normal component of electric field is

(8) $$\begin{array}{c} \Delta p_{n}=\frac{1}{2}\left\{\sqrt{\varepsilon_{0}^{2}\varepsilon_{1}^{2}+\frac{\sigma_{1}^{2}}{\omega^{2}}}E_{n,1}^{2}-\sqrt{\varepsilon_{0}^{2}\varepsilon_{2}^{2}+\frac{\sigma_{2}^{2}}{\omega^{2}}}E_{n,2}^{2}\right\}. \end{array}$$

Concerning the boundary condition (5), Δ*p*_*n*_ can finally be presented as

(9) $$\begin{array}{c} \Delta p_{n}=\frac{1}{2\omega }\left\{\sqrt{\varepsilon_{0}^{2}\varepsilon_{1}^{2}\omega^{2}+\sigma_{1}^{2}}-\frac{{\left(\varepsilon_{0}^{2}\varepsilon_{1}\varepsilon_{2}\omega^{2}+\sigma_{1}\sigma_{2}\right)}^{2}+\varepsilon_{0}^{2}\omega^{2}{\left(\varepsilon_{1}\sigma_{2}-\sigma_{1}\varepsilon_{2}\right)}^{2}}{{\left(\varepsilon_{0}^{2}\varepsilon_{2}^{2}\omega^{2}+\sigma_{2}^{2}\right)}^{3/2}}\right\}E_{n,1}^{2}. \end{array}$$

Both layers can be considered as ideal dielectrics when *ν*(*f*) = 2*πfε*_0_*ε*(*f*)/*σ*(*f*) ≫ 1; in this case, (9) reduces to Δ*p*_*n*_ = 1/2*ε*_1_/*ε*_2_*ε*_0_(*ε*_2_ − *ε*_1_) *E*_*n*,1_^2^. Contrarily, both layers are ideal conductors when *ν*(*f*) = 2*πfε*_0_*ε*(*f*)/*σ*(*f*) ≫ 1; in this case, (9) reduces to Δ*p*_*n*_ = 1/2*ωσ*_1_/*σ*_2_(*σ*_2_ − *σ*_1_)*E*_*n*,1_^2^.

Similar to this, the resultant pressure on the interface of two lossy dielectric layers produced by tangential component of electric field can be presented in the form

(10) $$\begin{array}{c} \Delta p_{t}=\frac{1}{2}\left\{\sqrt{\varepsilon_{0}^{2}\varepsilon_{2}^{2}+\frac{\sigma_{2}^{2}}{\omega^{2}}}-\sqrt{\varepsilon_{0}^{2}\varepsilon_{1}^{2}+\frac{\sigma_{1}^{2}}{\omega^{2}}}\right\}E_{t,1}^{2}. \end{array}$$

For two ideal dielectrics, Δ*p*_*t*_ = 1/2*ε*_0_(*ε*_2_ − *ε*_1_) *E*_*t*,1_^2^; for two ideal conductors, Δ*p*_*t*_ = 1/2*ω*(*σ*_2_ − *σ*_1_) *E*_*t*,1_^2^.

Total mechanical stress produced by RF current on the interface between two lossy dielectrics is Δ*p* = Δ*p*_*t*_ + Δ*p*_*n*_. From here, not only the conductivities and permittivities of two adjacent layers but also the direction of the current crossing the interface will define the value of generated electromechanical effect. Resultant mechanical stress on the interface will tend to move the layer having a higher permittivity into the layer having a lower permittivity, thus locally deforming the boundaries between the layers.

## 3. Electromechanical Effects Induced by RF Currents on EDJ and DSJ

While skin is a multilayer system containing more than three layers [1], we will further consider only EDJ and DSJ as the most important interfaces for skin aging. Electrical characteristics of SC, viable epidermis, dermis, and subcutis at 100 kHz and 1 MHz reported in [14–16] are summarized in Table 1.

Calculated values of parameter *ν*(*f*) = 2*πfε*_0_*ε*(*f*)/*σ*(*f*) describing the ratio between displacement and conductive current in different skin layers and in sWAT for RF of 100 kHz and 1 MHz are shown in Table 2. For RF of 100 kHz, displacement current dominates over the conductive current in SC and viable epidermis making these layers predominantly dielectric; contrarily, this ratio is low in dermis and sWAT, which means that at these frequencies, both layers are predominantly conductive.

For EDJ, layer 1 is viable epidermis and layer 2 is dermis. Pressures Δ*p*_*t*_ and Δ*p*_*n*_ induced on this interface at electrical field strength in epidermis of 100 V/cm are presented in Table 3. Of note, the tangential component of electric field of the same strength produces higher mechanical stress on this interface than the normal one.

For DSJ, layer 1 is dermis and layer 2 is sWAT. Pressures Δ*p*_*t*_ and Δ*p*_*n*_ induced on this interface at electrical fields in dermis of 100 V/cm are presented in Table 4. Of note, the normal component of electric field produces on this interface produces much higher mechanical stress than the tangential one.

It is seen that application of a low-frequency RF current can induce relatively high pressure of several kPa on DSJ tending to move dermis into sWAT and mechanically stimulating the cells located at this interface. This effect is frequency dependent, and stronger mechanical stress can be caused by application of a low-frequency of RF current.

## 4. Discussion

Application of RF current can induce mechanical stress on the interfaces between single layers in the skin as well as between dermis and subcutis, which are sufficiently involved in the maintenance of mechanical properties of the skin and are known to be strongly modified in aging. Two most prominent skin interfaces are EDJ and DSJ, containing papillary fibroblasts and dermal adipocytes, which are phenotypically and morphologically different from less reactive dermal fibroblasts and subcutaneous adipocytes, respectively. Mechanical stresses induced by RF current on these interfaces are strongly frequency dependent being higher by application of the low-frequency RF currents, and their values are high enough to stimulate the cells located near these interfaces and to induce their physiological reactions and morphological transformations. Of note, application of VHF-US can also induce a selective thermomechanical effect on the DSJ interface, but this effect increases with US frequency [9].

Mechanical modification of the skin interfaces is connected to modulation of caveolin-1 (Cav-1) expression. Cav-1 is a principle structural component of the caveola plasma membrane invaginations, which are present in almost all types of skin cells and adipocytes. This protein is strongly involved in rapid adaptation to cellular volume modifications, in multiple signal transduction processes, and in the processes of endo- and exocytosis as well as in intercellular adhesion. Modulation of Cav-1 can be provided both by a mild hyperthermia and/or mechanical stimulation, and it was considered as an important target in different inflammatory and hyperproliferative dermatological conditions as well as in prevention and therapy of skin aging [17, 18]. Moreover, Cav-1 is an important component of intercellular adhesion [18] and thus is strongly involved in mechanical properties of the cells and tissues. Its content can be modified yet by application of the low-level mechanical stresses of about 1 Pa [19], which are in the range of the pressures induced on the skin interfaces by RF currents (Tables 3 and 4), and this expression increases with increased stress. Modification of Cav-1 affects the adhesion between adjacent layers and thus influences the integral mechanical stability of the skin [1]. Sufficiently high mechanical stress produced on DSJ by application of low-frequency RF current can thus modify the structure of this interface and change the mechanical properties of the skin.

Mechanical effects of VHF-US and RF currents on the skin interfaces are frequency dependent. However, whereas interfacial mechanical stress induced by RF currents decreases with RF in the range between 0.1 MHz and 10 MHz that is mainly applied in dermatology and aesthetic medicine, an inverse frequency dependence should be observed for VHF-US for which both temperature gradient and mechanical stress on DSJ increase with frequency. Whereas future research in this field will be needed, we propose that interfacial therapy targeting EDJ and DSJ can be an effective method in antiaging and cellulite treatments.

## Figures and Tables

**Table 1:** Electrical conductivities and relative permittivities of different skin layers.

Tissue	0.1 MHz		1 MHz	
	**σ**, S/m	**ε**	**σ**, S/m	**ε**
SC	0.001	1,500	0.025	450
Epidermis	0.033	8,000	0.1	2,000
Dermis	1.0	15,000	1.0	600
sWAT	0.025	40	0.025	20

**Table 2:** Ratio *ν*(*f*) calculated for some RF in skin layers and sWAT.

Tissue	0.1 MHz	1 MHz
SC	8.6	1.0
Epidermis	1.35	1.11
Dermis	0.08	0.03
sWAT	0.009	0.045

**Table 3:** Pressure on DEJ induced by RF current.

RF	Δ*p*_*t*_, Pa	Δ*p*_*n*_, Pa
0.1 MHz	78.9	4.1
1 MHz	7.66	1.0

**Table 4:** Pressure on DHJ induced by RF current.

RF	Δ*p*_*t*_, Pa	Δ*p*_*n*_, Pa
0.1 MHz	81.0	3,500
1 MHz	8.6	13

## Data Availability

All the data are present in the article. No additional data exist.
